# Argonaute protein-based nucleic acid detection technology

**DOI:** 10.3389/fmicb.2023.1255716

**Published:** 2023-09-08

**Authors:** Zhiyun Wu, Li Yu, Weifeng Shi, Jinhong Ma

**Affiliations:** ^1^Department of Clinical Laboratory, The Third Affiliated Hospital of Soochow University, Changzhou, Jiangsu, China; ^2^Jiangsu Key Laboratory of Medical Science and Laboratory Medicine, Institute of Stem Cell, School of Medicine, Jiangsu University, Zhenjiang, China

**Keywords:** nucleic acid detection technology, pathogen diagnosis, Argonaute, nucleases, biosensor

## Abstract

It is vital to diagnose pathogens quickly and effectively in the research and treatment of disease. Argonaute (Ago) proteins are recently discovered nucleases with nucleic acid shearing activity that exhibit specific recognition properties beyond CRISPR–Cas nucleases, which are highly researched but restricted PAM sequence recognition. Therefore, research on Ago protein-mediated nucleic acid detection technology has attracted significant attention from researchers in recent years. Using Ago proteins in developing nucleic acid detection platforms can enable efficient, convenient, and rapid nucleic acid detection and pathogen diagnosis, which is of great importance for human life and health and technological development. In this article, we introduce the structure and function of Argonaute proteins and discuss the latest advances in their use in nucleic acid detection.

## Introduction

Nucleic acid detection technology is an essential tool for molecular diagnosis, with crucial applications in disease diagnosis, treatment, and biosecurity. Thus, it is vital to develop nucleic acid detection technology that is simple to use, accurate, specific, and cost-effective. Currently, commonly employed diagnostic methods include polymerase chain reaction (PCR), antigen–antibody reactions, and tissue biopsies. However, PCR, the most widely used nucleic acid amplification technique, faces challenges with nonspecific amplification. To improve the precision of nucleic acid detection, innovative approaches are needed. The discovery and application of programmable nucleases, such as zinc-finger nucleases (ZFNs) (Wu et al., 2007; Shamshirgaran et al., 2022), transcription activator-like effector nucleases (TALENs) (Zhang et al., 2019), and clustered regularly interspaced short palindromic repeat systems (CRISPR-associated nuclease) (Mojica et al., 2009), have revolutionized diagnostic techniques.

Zinc finger nucleases (ZFNs) are the first generation of programmable restriction endonucleases and consist of a zinc finger DNA recognition binding part formed by multiple zinc finger units in tandem and a DNA cleavage part of the restriction endonuclease *Fok* I. They can generate a cut at a specific site by recognizing a particular sequence of DNA, which is then repaired using the cell’s inherent homologous recombination repair mechanism (Wu et al., 2007). Transcription activator-like effector nucleases (TALENs) are proteases inspired by ZFNs. Researchers have replaced the zinc finger recognition binding site with a transcription activator effector protein (TALE) to achieve the same recognition cleavage function (Zhang et al., 2019). Clustered regularly interspaced short palindromic repeats (CRISPR) and their associated proteins (Cas) can be recognized by PAM sequences, which are recognized and then inserted between the leader sequence and the interspaced repeats and subsequently transcribed into crRNA, which forms a complex with the Cas protein to recognize and act as a cleavage (Mojica et al., 2009; Li et al., 2019). CRISPR-Cas systems have evolved rapidly in recent years (Wang et al., 2020) and have been used in sensitive and specific pathogen nucleic acid detection and gene editing, with the development of nucleic acid detection platforms such as Cas12-DETECTOR (Broughton et al., 2020), Cas14-DETECTOR (Aquino-Jarquin, 2019), Cas9-FLASH (Kellner et al., 2019; Quan et al., 2019) and Cas13-SHERLOCK (Aman et al., 2020). SHERLOCK detection is highly specific for single nucleotide mutations and has been shown to discriminate between Zika virus SNPs (Pardee et al., 2016). Proteins such as Cas12 and Cas13 have also been used in viral detection for SARS-CoV-2 (Ding et al., 2020; Marais et al., 2020; Rabe and Cepko, 2020; Wozniak et al., 2020; Nouri et al., 2021). Apart from several programmable nucleases described above, there are several other nucleases. For example, because of their extremely specific DNA identification and nucleic acid endonucleating activity, meganucleases that are found in cellular mitochondria and chloroplasts have been developed into artificial nucleases (Maeder and Gersbach, 2016; Khalil, 2020). Besides, nucleases like S1 nuclease (Filer et al., 2019) can be utilized in nucleic acid assays to safeguard the target nucleic acid, break down unbound probes, and other heterogeneous nucleic acids. Nucleases can also be involved in the repair of clip mismatches (Tsutakawa et al., 2014). Furthermore, nucleases, such as DNAzyme (He et al., 2014), Exo III (Xu et al., 2017), can be involved in the reaction as tools for auxiliary signal amplification (Gerasimova and Kolpashchikov, 2014; Hong et al., 2017). Nucleases not only appear as a tool in the detection process, but can also be viewed as the object under detection. They have the potential to be used as a biomarker for the detection and diagnosis of clinical infections due to changes in their catalytic capacity and preferred substrate (Garcia Gonzalez and Hernandez, 2022). The recently discovered Argonaute protein (Ago) is also a class of nucleic acid endonucleases that use nucleic acids as guides and has been heavily investigated by researchers to develop more efficient diagnostic platforms for nucleic acid detection.

## Argonaute nucleases

Argonaute proteins were first mentioned in a study of *Arabidopsis* mutants in 1998 and named Argonaute because of the curly leaves of the mutant plants, which resemble the tentacles of a squid (Bohmert et al., 1998). Ago protein family members are widely distributed in various organisms and can be classified according to their origin as eukaryotic Ago proteins (eAgos) and prokaryotic Ago proteins (pAgos). In general, eAgos are more structurally uniform and exhibit high conservation that only mediates RNA-directed RNA interference. In contrast, pAgos exhibit more structural and functional diversity (Sheng et al., 2014). And some bacteria and archaea Argonaute proteins can participate in host defense processes by interfering with foreign nucleic acid invasion, such as against DNA viruses and foreign plasmids (Swarts et al., 2014).

**Figure 1:**
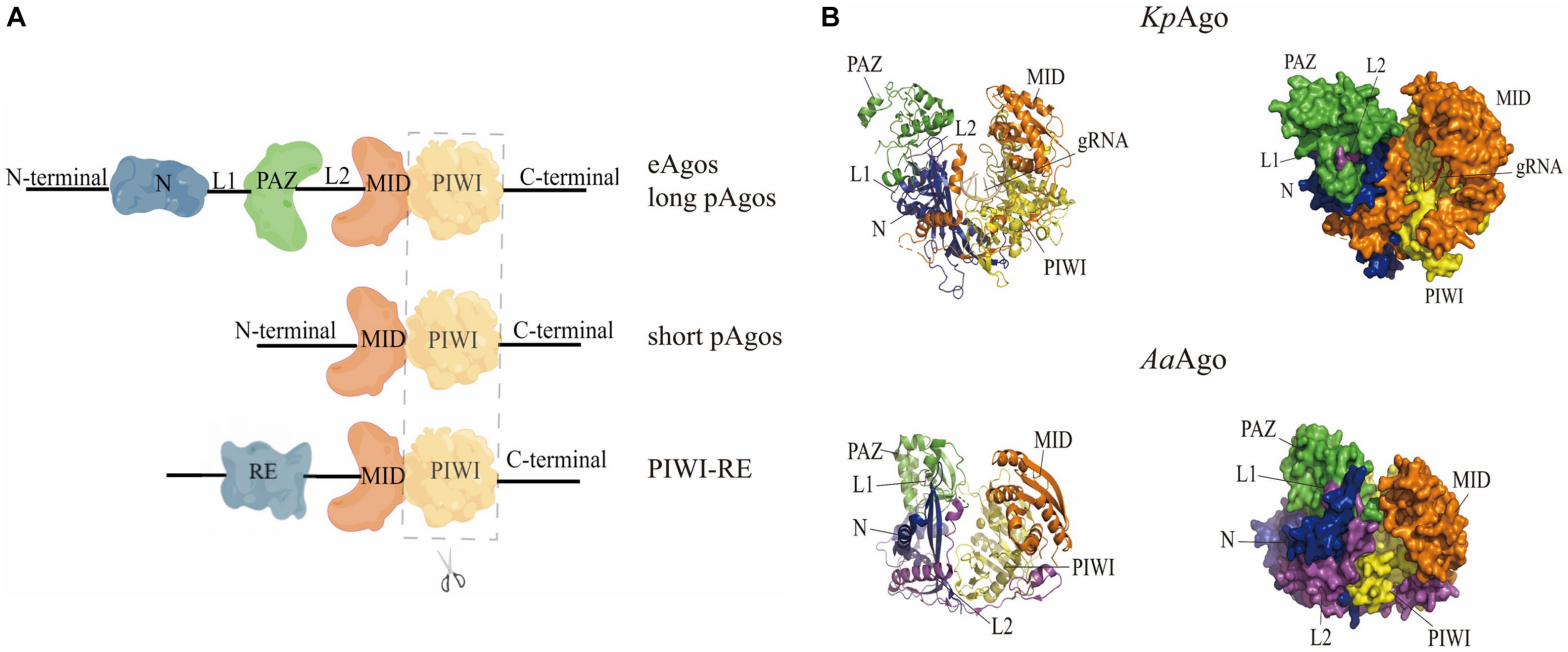
Schematic diagram of the structure of the Ago protein. **(A)** Linear representations of Ago protein structures. **(B)** Schematic structures of *Kp*Ago (eAgo) and *Aa*Ago (pAgo), including N (blue), L1 linker (purple), PAZ (green), L2 linker (purple), MID (orange), PIWI (yellow).

The Ago protein family is broadly similar in structure (Figure 1). pAgos can be divided into three categories according to their structural length: long pAgos, short pAgos, and PIWI-RE proteins (prokaryotic PIWI with only conserved R and E residues). The structure of long pAgos is very similar to that of eAgos and they both form a two-lobed scaffold (Kaya et al., 2016). The N-terminal lobe is connected with the PAZ domain by the L1 linker, and the C-terminal lobe is composed of the MID domain, the PIWI domain and the C-terminal. Finally, the L2 linker connects the two lobes. The structures of the short pAgos and PIWI-RE proteins are similar to that of the long pAgos at the C-terminal, except that the N-terminal end is more straightforward, without the PAZ structural domain and the L1 and L2 linker connections (Kaya et al., 2016; Sheng et al., 2017; Lisitskaya et al., 2018).

**Figure 2:**
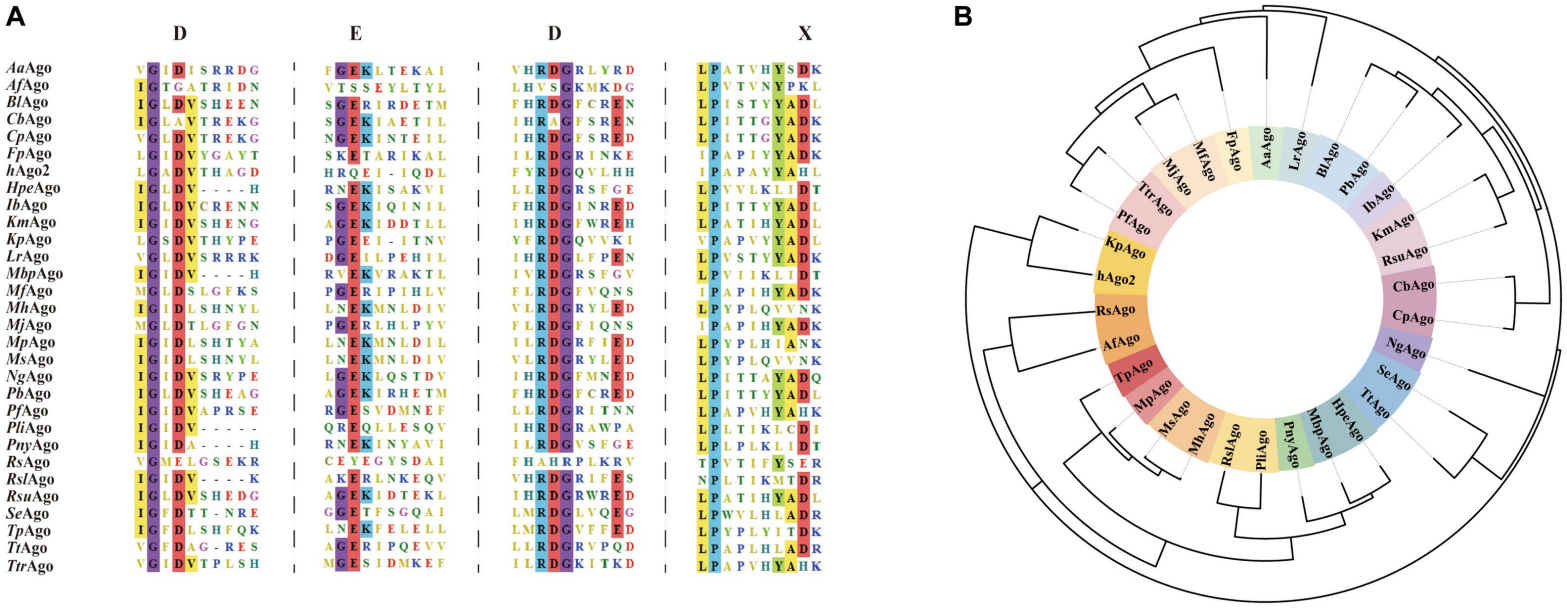
**(A)** Multiple sequence alignments of Argonaute proteins. The sequences include *Aa*Ago (*Aquifex aeolicus* Argonaute, WP_010880937.1), *Af*Ago (*Archaeoglobus fulgidus* Argonaute, WP_010878815.1), *Bl*Ago (*Brevibacillus laterosporus* Argonaute, WP_277546270.1), *Cb*Ago (*Clostridium butyricum* Argonaute, WP_058142162.1), *Cp*Ago (*Clostridium perfringens* Argonaute, WP_003477422.1), *Fp*Ago (*Ferroglobus placidus* Argonaute, WP_012966655.1), *h*Ago2 (*Homo sapiens* Argonaute, NP_036286.2), *Hpe*Ago (*H. penzbergensis* Argonaute, WP_139173808.1), *Ib*Ago (*Intestinibacter bartlettii* Argonaute, WP_007287731.1), *Km*Ago (*Kurthia massiliensis* Argonaute, WP_010289662.1), *Kp*Ago (*Vanderwaltozyma polyspora* Argonaute, XP_001644461.1), *Lr*Ago (*Limnothrix rosea* Argonaute, WP_075892274.1), *Mbp*Ago (*Mucilaginibacter paludism* Argonaute, WP 008504757.1), *Mf*Ago (*Methanocaldococcus fervens* Argonaute, WP_015791216.1), *Mh*Ago (*Marinitoga hydrogenitolerans* Argonaute, UniProtKBSwiss-Prot A0A1M5A5Z8.1), *Mj*Ago (*Methanocaldococcus jannaschii* Argonaute, WP_010870838.1), *Mp*Ago (*Marinitoga piezophila* Argonaute, WP_014295921.1), *Ms*Ago (*Marinitoga* sp. 1155 Argonaute, WP_047265940.1), *Ng*Ago (*Natronobacterium gregoryi* Argonaute, WP_005580376.1), *Pb*Ago (*Paenibacillus borealis* Argonaute, WP_042211195.1), *Pf*Ago (*Pyrococcus furiosus* Argonaute, WP_011011654.1), *Pli*Ago (*Pseudooceanicola lipolyticus* pAgo, WP_100161590.1), *Pny*Ago (*Pedobacter nyackensis* Argonaute, WP_084286803.1), *Rs*Ago (*Rhodobacter sphaeroides* Argonaute, UniProtKB A4WYU7), *Rsl*Ago (*R. slithyformis* Argonaute, WP_013921749.1), *Rsu*Ago (*Rummeliibacillus suwonensis* Argonaute, WP_146547607.1), *Se*Ago (*Synechococcus elongatus* Argonaute, WP_011244830.1), *Tp*A*go* (*Thermotoga profunda* Argonaute, WP_041081268.1), *Tt*Ago (*Thermus thermophilus* Argonaute, WP_011174533.1), *Ttr*Ago (*Thermococcus thioreducens* Argonaute, WP_055429304.1). The catalytic tetrad DEDX of Argonautes is highlighted red in the sequence alignment. **(B)** Schematic phylogenetic tree of above described Argonaute proteins.

It has been shown that the PAZ, MID, and PIWI domains are very important parts of Argonaute protein function. PAZ and MID domains form a binding pocket that anchors and binds to the 3′ and 5′ ends of the target strand, and the target is catalyzed by the PIWI domain for shearing (Song et al., 2004; Yuan et al., 2005). The PAZ domain contains conserved aromatic residues and forms a fold that guides and binds the target DNA/RNA fragments (Cao et al., 2019), but this domain is not necessarily required for the shearing properties of Argonaute proteins. A team found that *Af*Ago (Ago from *Archaeoglobus fulgidus*), which is a short pAgos, consists of an N-terminal, MID and PIWI structural domain. *Af*Ago without a PAZ structural domain can also recognize and bind through the channels formed by the MID and PIWI structural domains, and perhaps the absence of the PAZ domain has an impact on binding affinity (Yan et al., 2003; Yuan et al., 2005). The PIWI domain is structurally and functionally similar to ribonuclease H (RNase H). The PIWI domain is capable of exerting nucleic acid endonuclease activity to hydrolyze RNA phosphodiester bonds and specifically catalyze the cleavage of target genes (Hur et al., 2013). This catalytic activity is achieved through the DEDX (X = N, D, H) tetrameric structure (Figure 2A), such as the DEDD tetramer present in the PIWI structural domain of *Cp*Ago (Ago from *Clostridium perfringens*) found in the study, and this cleavage activity was lost with the mutation at position D614 (Parker et al., 2005). In order to gain a comprehensive understanding of the genetic relationship and characteristics of well-studied pAgos, Figures 2A,B depict the conserved active sites and a phylogenetic tree of recently studied Argonaute proteins, respectively. A summary of the biochemical properties and structural information can be found in Table 1. The primary focus of research has been on bacterial and archaeal Argonautes, which exhibit nuclease activity and primarily recognize DNA targets, unlike their eukaryotic counterparts that utilize RNA guides and targets within cells. However, some prokaryotic Argonautes, such as *Pli*Ago and *Pny*Ago, can also cleave RNA targets, albeit with lower efficiency (Lisitskaya et al., 2022). While most prokaryotic Argonautes bind DNA guides, a few, like *Mp*Ago from *Marinitoga piezophila* and its closest homologs, show a preference for RNA guides (Kaya et al., 2016). The functional significance of this phenomenon and the specific structural features responsible for distinguishing RNA/DNA guides and targets in prokaryotic Argonautes remain unknown (Wang et al., 2008; Kropocheva et al., 2021). It is hypothesized that these features are primarily influenced by variations in the structures of the MID and PIWI domains. Further studies investigating the activity and structures of prokaryotic Argonaute proteins are necessary to address these questions.

**Table 1:** Properties of Argonaute protein with shear activity.

Host	Argonaute, protein ID	PDB code	Molecular weights (kD)	Guide, length (nt)^а^	5′ end of the guide^b^	Target	Reaction temperature^с^	Ion	Catalytic activity	References
*Aquifex aeolicus*	*Aa*Ago, WP_010880937.1	2F8T	83.12	DNA, 18–24	5′-P	RNA, (DNA-)	opt. 55°C; up to 75°C	Mg^2+^, Mn^2+^	Guide-dependent	Yuan et al. (2005, 2006) and Rashid et al. (2007)
*Archaeoglobus fulgidus*	*Af*Ago, WP_010878815.1	1YTU	49.24	RNA	–	RNA	69–85°C	–	Guide-dependent	Jin et al. (2021)
*Brevibacillus laterosporus*	*Bl*Ago, WP_277546270.1	–	80.43	DNA, 14–35	5′-P; 5′-OH	DNA	10–95°C, opt. 65°C	Mg^2+^, Mn^2+^, Co^2+^, Ni^2^	Guide-dependent	Dong et al. (2021)
*Clostridium butyricum*	*Cb*Ago, WP_058142162.1; *Cbc*Ago, WP_045143632.1	6QZK (bound to a guide DNA (5' deoxycytidine) and a 19-mer target DNA)	85.98; 85.86	DNA (RNA weaky) 16–18	5′-P	DNA (RNA weakly)	opt. 37°C; 30–60°C (opt. 37–42°C; 25–55°C)	Mg^2+^, Mn^2+^, Co ^2+^	Guide-dependent; guide-independent	García-Quintans et al. (2019), Hegge et al. (2019), and Kuzmenko et al. (2019, 2020)
*Clostridium perfringens*	*Cp*Ago, WP_003477422.1	–	85.61	DNA, 15–30 (≥12)	5′-P	DNA (RNA)	opt. 37°C; 4–70°C	Mg^2+^, Mn^2+^	Guide-dependent	Cao et al. (2019)
*Ferroglobus placidus*	*Fp*Ago, WP_012966655.1	–	91.45	DNA, 15–16	5′-P	DNA	75–99°C	Mg^2+^, Mn^2+^, Co^2+^, Ni^2+^	Guide-dependent	Guo et al. (2021)
*Hydrobacter penzbergensis*	*Hpe*Ago, WP_139173808.1	–	91.29	DNA, 16–20	5′-P; 5′-OH	RNA	30–44°C	Mg^2+^, Mn^2+^	Guide-dependent	Lisitskaya et al. (2022)
*Intestinibacter bartlettii*	*Ib*Ago, WP_007287731.1	–	85.99	DNA, 15–30 (≥14)	5′-P	DNA	opt. 37°C; 4–60°C	Mg^2+^, Mn^2+^	Guide-dependent	Cao et al. (2019)
*Kurthia massiliensis*	*Km*Ago, WP_010289662.1	–	85.36	DNA (RNA), 16–20 (≥12)	5′-P	DNA (RNA)	opt. 37–60°С; from 25°С	Mn^2+^, Mg^2+^, weakly Co^2+^	Guide-dependent; guide-independent (37°С)	Kropocheva et al. (2021) and Liu et al. (2021)
*Limnothrix rosea*	*Lr*Ago, WP_075892274.1	–	83.09	DNA, 16–18 (≥10)	5′-P	DNA	opt. 37°C; 30–54°C	Mn^2+^, Mg^2+^, weakly Co^2+^	Guide-dependent; guide-independent	Kuzmenko et al. (2019)
*Mucilaginibacter paludism*	*Mbp*Ago, WP_008504757.1	–	91.79	DNA, 15–17	5′-P; 5′-OH	RNA	0–65°C; opt. 20–65°C	Mn^2+^, Mg^2+^	Guide-dependent	Li et al. (2022)
*Methanocaldococcus fervens*	*Mf*Ago, WP_015791216.1	–	83.54	DNA, RNA, 16	5′-P DNA and RNA; 5′-OH DNA	DNA	opt. 80–90°C; from 54°C	Mn^2+^, Mg^2+^, Co ^2+^	Guide-dependent	Willkomm et al. (2017)
*Marinitoga hydrogenitolerans*	*Mh*Ago, WP_072865986.1	–	76.71	DNA, 14–21 RNA, 18–21	5′-P; 5′-OH	DNA, RNA	37–65°C; opt. 60–65°C	Mn^2+^, Mg^2+^	Guide-dependent	Wang et al. (2022)
*Methanocaldococcus jannaschii*	*Mj*Ago, WP_010870838.1	5G5T	84.58	DNA, 15–41	5′-P	DNA	opt. 75–95°C; from 37°C	Mg ^2+^	Guide-dependent; guide-independent	Zander et al. (2014, 2017) and Willkomm et al. (2017)
*Marinitoga piezophila*	*Mp*Ago, WP_014295921.1	5I4A (complexed with 5’-OH guide RNA)	75.91	RNA, 16–40	5′-OH	DNA (RNA)	37, 55, 60°C	Mn^2+^, Mg^2+^, weakly Ni^2+^	Guide-dependent	Kaya et al. (2016), Doxzen and Doudna (2017), and Lapinaite et al. (2018)
*Marinitoga* sp.*1155*	*Ms*Ago, WP_047265940.1	–	76.64	RNA	–	DNA	–	–	–	Jin et al. (2021)
*Natronobacterium gregoryi*	*Ng*Ago, WP_005580376.1	5I4A (complexed with 5’-OH guide RNA)	98.26	RNA, 16–40	5′-OH	DNA (RNA)	37°C	Mn^2+^, Mg^2+^, weakly Ni^2+^	Guide-dependent	O'Geen et al. (2018), Qi et al. (2016), and Wu et al. (2017)
*Paenibacillus borealis*	*Pb*Ago, WP_042211195.1	–	80.77	DNA, 14–35	5′-P; 5′-OH	DNA	10–95°C, opt. 37°C	Mn^2+^, Mg^2+^	Guide-dependent	Dong et al. (2021)
*Pyrococcus furiosus*	*Pf*Ago, WP_011011654.1	1Z26	90.39	DNA, 15–31	5′-P	DNA	opt. 87–99.9°C; from 37°C	Mn^2+^, Co ^2+^	Guide-dependent; guide-independent	Song et al. (2004) and Swarts et al. (2015)
*Pseudooceanicola lipolyticus*	*Pli*Ago, WP_100161590.1	7R8I	87.92	DNA, 14–20	5′-P; 5′-OH	RNA	30–44°C	Mn^2+^, Mg^2+^	Guide-dependent	Lisitskaya et al. (2022)
*Pedobacter nyackensis*	*Pny*Ago, WP_084286803.1	–	90.17	DNA, 16–20	5′-P; 5′-OH	RNA	30–44°C	Mn^2+^, Mg^2+^	Guide-dependent	Lisitskaya et al. (2022)
*Runella slithyformis*	*Rsl*Ago, WP_013921749.1	–	92.61	DNA, 14–20	5′-P	RNA	30–44°C	Mn^2+^, Mg^2^	Guide-dependent	Lisitskaya et al. (2022)
*Rummeliibacillus suwonensis*	*Rsu*Ago, WP_146547607.1	–	85.60	DNA, RNA (weakly), 15–25	5′-P; 5′-OH	DNA, RNA	opt. 25–60°C	Mn^2+^, Mg^2^	Guide-dependent	Jiang et al. (2022)
*Synechococcus elongatus*	*Se*Ago, WP_011244830.1	6KIG	83.65	DNA, 14–24	5′-P	DNA	opt. 37°C	Mn^2+^, weakly Mg^2+^	Guide-dependent; guide-independent	Olina et al. (2020) and Cao et al. (2020)
*Thermotoga profunda*	*Tp*Ago, WP_041081268.1	–	75.06	RNA, 21	5′-OH	DNA	60°C	Mg^2+^, Mn^2+^	Guide-dependent	Kaya et al. (2016)
*Thermus thermophilus*	*Tt*Ago, WP_011174533.1	4NCB	76.61	DNA, 13–25	5′-P	DNA (RNA)	opt. 73–75°C	Mg^2+^, Mn^2+^	Guide-dependent; guide-independent	Wang et al. (2008, 2009), Sheng et al. (2014, 2017), and Swarts et al. (2017)
*Thermococcus thioreducens*	*Ttr*Ago, WP_055429304.1	–	87.36	DNA, ≥14	5′-P; 5′-OH	DNA	65–95°C; opt. 75–95°C	Mg^2+^, Co^2+^, Mn^2+^	Guide-dependent; guide-independent	Fang et al. (2022)

^a^ The type of guide nucleic acid and its length (number of nucleotides).

^b^ Characteristics of the 5′-end of the guide: P-phosphate group, OH-hydroxyl group; Here and further: –unknown.

^c^ opt.-the optimum reaction temperature, the temperature range at which the Argonaute cleaves targets is also given Discrete temperature values are given for publications where measurements were carried out only under these conditions.

The Ago protein is also found to play an essential role in the formation of the RNA silencing complex (RISC), as a critical protein in the silencing pathway, and is bound to another class of glycine- and tryptophan-rich (GW/WG) proteins known as the “Ago hook,” which are involved in maintaining the maintenance of genomic stability (Zander et al., 2014), such as *Rs*Ago (Ago from *Rhodobacter sphaeroides*), which was discovered to bind to foreign nucleic acids and thus perform host defense (Hegge et al., 2019).

## Argonaute protein-based nucleic acid detection platform

While polymerase chain reaction (PCR) has long reigned in molecular diagnostics in recent years, people have urgent demands, such as the SARS-CoV-2 pandemic, for newer technologies that can provide more direct and convenient detection methods. This inspires researchers to explore new molecular diagnostic methods. Researchers have combined CRISPR-Cas or Argonaute proteins with biosensing technologies such as fluorescence and microfluidic control devices to develop new nucleic acid detection technologies (Santiago-Frangos et al., 2022). Nucleic acid biosensing systems such as DETECTR and HOLMES have been established based on CRISPR-Cas12a and CRISPR-Cas9 to meet various clinical testing requirements (Jiang and Doudna, 2017; He et al., 2019). At the same time, these new biosensing platforms also show good sensitivity and specificity. Similar to Cas proteins, Argonaute proteins can recognize and cleave target sequences under the guidance of gDNA. However, this kind of protein requires no additional specific sequences (such as PAM sequences) to recognize the target sequences, and the proteins are widely found in a variety of organisms (Kropocheva et al., 2022). Based on this, researchers have developed a range of nucleic acid detection platforms.

## DNA-targeted nucleic acid detection platforms

*Pf*Ago (*Pyrococcus furiosus* Argonaute), a DNA-guided nucleic acid endonuclease derived from thermophilic archaea with an optimal temperature between 80 and 100°C, is the first Ago protein with a complete three-dimensional structure. It is reckoned that *Pf*Ago uses the 15–31 nt 5′ phosphorylation of siDNAs to shear ssDNA. The optimal temperature for this shearing activity is 87–99°C. The reaction requires the involvement of Mn^2+^, Mg^2+^, and preferably the NaCl concentration of 50–250 mM (Swarts et al., 2015; He et al., 2021).

A research team established a novel nucleic acid detection platform named PAND (*Pf*Ago-mediated Nucleic acid Detection) (He et al., 2019). The platform combines PCR with *Pf*Ago, which has the DNA-targeted nucleic acid shearing property, to enable multiplexed nucleic acid detection of DNA. The recreation uses the three given gDNA sequences to instruct *Pf*Ago to perform nucleic acid shearing, which has shown high specificity for clinical samples. In 2021, Wang’s team used the PAND platform in the detection of SARS-CoV-2 (Wang et al., 2021), and recently, Chen’s team used it in the detection of *Parvovirus* B19 (B19V) (Chen et al., 2023). Based on the PAND detection platform, researchers have chosen to build a PLCR detection platform by combining ligase chain reaction (LCR) with *Pf*Ago, increasing the specificity for distinguishing single base mutations in gene sequences and requiring only two temperature shifts to complete the amplification cycle (Wang et al., 2021). Compared to PAND, PLCR uses a modified LCR product for gDNA, rather than adding additional gDNA after the target amplification, which also greatly simplifies the equipment requirements. It is noteworthy that amplification of non-target sequences should be avoided by controlling the number of cycles and Tm value conditions. LCR reactions are usually performed under conditions such as 30 cycles and oligonucleotides with Tm values close to 50°C, which avoid amplification independent of the template. This platform can reach detection limits of 10 aM in 70 min and 1 aM in 100 min. To some extent, this method achieves the accuracy comparable to that of qPCR in a much shorter time, enabling susceptible, multichannel, precise single-base mutant detection (Liu et al., 2021; Wang et al., 2021).

Based on the high activity exhibited by *Pf*Ago at 95°C, this nucleic acid biosensing platform was combined with usPCR to establish a USPCRP platform. This method amplifies the target nucleic acid in the sample by PCR to obtain the appropriate guide DNA, thus reducing the time and human resources from designing the gDNA. This also allows the USPCRP system to show higher sensitivity, up to 10 aM, and high specific single-base resolution (He et al., 2019, 2021). In order to reduce the annealing temperature and the cycle time, the length of the double-stranded DNA product should be designed to be 22–28 bp, and two primers need to be designed: prime-g and prime-h. Primer-g is phosphorylated at the 5′ end and is used to generate the guide DNA from the target DNA. Primer-h is the conventional primer for higher yields. Surprisingly, the study mapped the temperature and time of denaturation and annealing in PCR and found that at a temperature combination of 70°C/40°C, substantial yields of guide DNA could be obtained in 30 min at a time as short as 1 s, which yielded a substantial time benefit (He et al., 2021).

**Figure 3:**
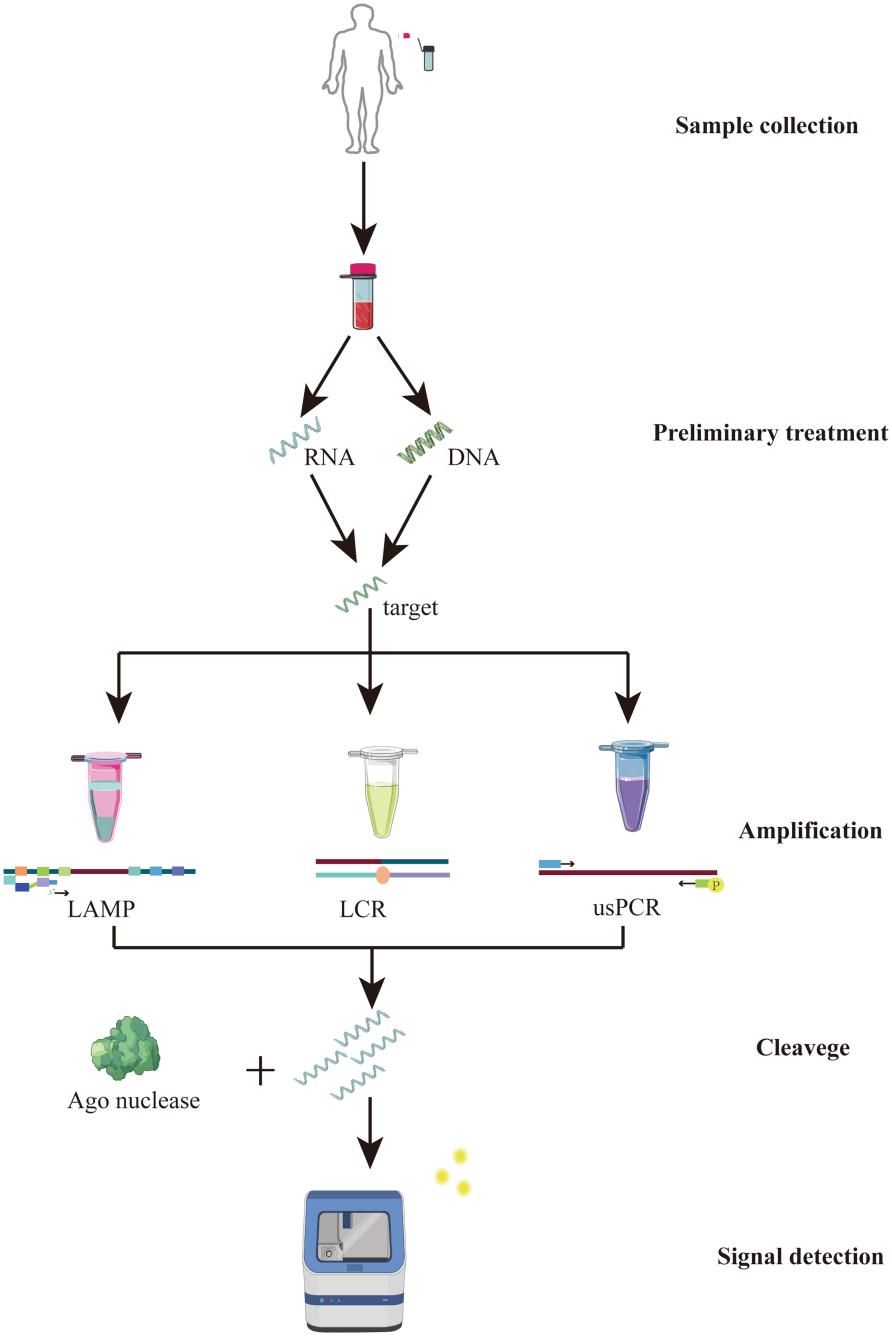
Schematic illustration of the *Pf*Ago-based MULAN, PLCR and USPCRP detection system.

At the same time, another one-pot multiplex detection platform, MULAN (Multiplex Argonaute-based Nucleic acid detection system), also based on *Pf*Ago, was developed. It is a platform that integrates the amplification-cleavage-assay steps in a single platform for portable, sealed-tube operation, avoiding the risk of splitting and contamination. The specific cleavage properties of the *Pf*Ago protein are exploited to increase the amplification product and fluorescence signal by designing two corresponding gDNAs for the amplification target. Primary cleavage of one strand of the gDNA generates a new gDNA that directs secondary specific division with different fluorescence modifications. The presence of the target nucleic acid sequence is determined by detecting the fluorescence signal from various channels. In addition, this single-tube system contains a separate space that allows amplification products to be mixed with *Pf*Ago, avoiding contamination from exposure to the external environment. This mini isothermal fluorescence detector (MIFD) design makes the whole operation more convenient, providing a new avenue for POCT. More notably, this device enables the simultaneous detection of multiple viruses by loading three sets of primers, gDNA, and fluorescent agents designed for SARS-CoV-2, Influenza A virus, and Influenza B virus multiplexes into a single jar of the detection system. In the absence of observed cross-reactivity, the MULAN system can specifically produce fluorescent signals for the respective targets, and no dissimilar signs are observed for viruses that are not present (Li et al., 2022; Ye et al., 2022). This result indicates that the MULAN system can achieve accurate viral multiplex detection in the presence of a single Ago protease. The schematic of MULAN、PLCR and USPCRP system was shown in Figure 3. CRISPR detection systems have also been successful in detecting different targets in a single reaction, but each requires an extra Cas protein. This reflects the great promise of Ago proteins in the development of nucleic acid detection technologies (Gootenberg et al., 2018; Myhrvold et al., 2018; Ooi et al., 2021). Recently, a team studying *Enterocytozoon hepatopenaei* (EHP) combined *Pf*Ago with recombinase polymerase amplification (RPA) to establish a method for the detection of pathogenic bacteria. This method has a satisfactory performance in that it does not require strict laboratory conditions, which provides a new method for the multiplex identification of pathogenic bacteria (Yang et al., 2023).

In addition to developing *Pf*Ago for use in nucleic acid detection platforms, other Ago proteins are being increasingly exploited. Mengjun Fang’s team mined a new Argonaute from the archaeon *Thermococcus thioreducens* (*Ttr*Ago) and used it for the detection of HBV DNA. Another *Tt*Ago-based platform named NAVIGATER (Nucleic Acids of Clinical Interest Via DNA-Guided Argonaute from *Thermus thermophilus*) has been developed and used in the detection of single nucleotide mutations by designing a guide complementary to the predominant wild-type nucleic acid sequence in the sample (Song et al., 2020). The nuclease activity of *Tt*Ago declines when a rare mutation occurs at position 10 or 11 of the target nucleic acid. The researchers designed a guide sequence complementary to the predominant wild-type nucleic acid sequence in the sample. The number of mutants increases when detected and the wild-type target nucleic acid sequence is cleaved by Argonaute, allowing the detection of the presence of mutants. Then SNVs were enriched by PCR amplification, and this method can specifically enrich multiple mutant alleles in a single sample. Compared to previously reported rare allele enrichment methods, NAVIGATER exhibits great strengths. For example, *Tt*Ago behaves flexibly because it does not require a PAM motif or a specific recognition site and a single *Tt*Ago-guide complex can cleave several targets (Sternberg et al., 2014; Swarts et al., 2014).

## RNA-targeted nucleic acid detection platforms

During RNA detection, it is also possible to link *Pf*Ago to the ligase chain reaction (RT-LCR), which requires an additional step in reverse transcription during the reaction of PLCR. However, this method does not require RNA for guidance, avoids the instability and higher costs associated with RNA, and allows for multichannel detection. In a trial of clinical samples, HPV genomic DNA from a cervical swab and SARS-CoV-2 genomic RNA from a throat swab were tested in separeate channels. The N gene and the ORF 1ab gene of SARS-CoV-2 and HPV subtypes were tested separately, and it has been found that this method could achieve high sensitivity for DNA or RNA with a sensitivity of up to 10 aM in 70 min. Although absolute quantification is not yet possible, the advantages of sensitivity, specificity, and high channelization are already considerable (Wang et al., 2021).

A team has developed an isothermal nucleic acid detection platform for RNA using the nucleic acid detection properties of the pAgo protein in combination with a reverse transcription reaction, named MAIDEN (Mesophilic Ago-based Isothermal Detection method) (Ye et al., 2022). The target nucleic acid sequence is reverse transcribed by reverse transcriptase. The RNA in the DNA–RNA hybrid strand produced by reverse transcription is hydrolyzed with RNase H, and the resulting ssDNA is used for recognition. The secondary gDNA is produced by reacting the designed primary gDNA with the Ago-gDNA complex. The ssDNA is cleaved by recognition of the Ago-secondary gDNA complex. The experiments showed that the reverse transcription reaction could be simplified with RNase H hydrolysis digestion. The reverse transcription process could be carried out simultaneously with the Ago stepwise cleavage reaction under the same moderate temperature conditions, which further simplified the reaction process. *Km*Ago from the thermophilic bacterium *Kurthia massiliensis*, combined with a 16–20 nt long 5′-phosphorylation-guided nucleic acid sequence capable of specific cleavage of DNA/RNA target sequences under DNA/RNA guidance (Kropocheva et al., 2021). Recently, researchers have used *Km*Ago (Ago from *Kurthia massiliensis*) and *Pb*Ago (Ago from *Paenibacillus borealis*) in this detection system and have achieved entirely satisfactory detection limits in a one-pot assay, with detection limits of 4.0 nM for *Km*Ago and 12.5 nM for *Pb*Ago (Li et al., 2022). However, compared to thermophilic *Pf*Ago, the activity of mesophilic *Km*Ago is low and a large number of enzymes need to be added in the experiment. In the future, it is necessary to apply a protein engineering strategy to improve the activity of mesophilic *Km*Ago at moderate temperatures to improve the sensitivity of mesophilic Ago-based detection.

Combining *Pf*Ago with reverse transcriptase-based isothermal amplification (RT-LAMP) has resulted in a rapid, portable assay system (SPOT) (Xun et al., 2021). This system consists of an assay system capable of precisely controlled temperature and fluorescence detection and a device capable of a mobile power supply. The system involves optimizing the GC content and length of the reagents to reduce unwanted background fluorescence and separates the LAMP and *Pf*Ago reaction components with paraffin wax. This convenient and all-in-one assay system offers a new approach to responding to and deploying large-scale epidemics and does not require a large number of specialist personnel, requiring only simple sample pre-processing to operate. Changjing Yuan’s team developed an isothermal amplification detection platform based on *Tt*Ago, called the *Thermus thermophilus* Argonaute-based thermostable exponential amplification reaction (*Tt*AgoEAR), which has shown rewarding reliability and sensitivity for the detection of viral RNA, lncRNA, and mRNA in various specimens (Yuan et al., 2023). This platform can detect RNA targets at 66°C with attomolar sensitivity and single-nucleotide resolution.

**Figure 4:**
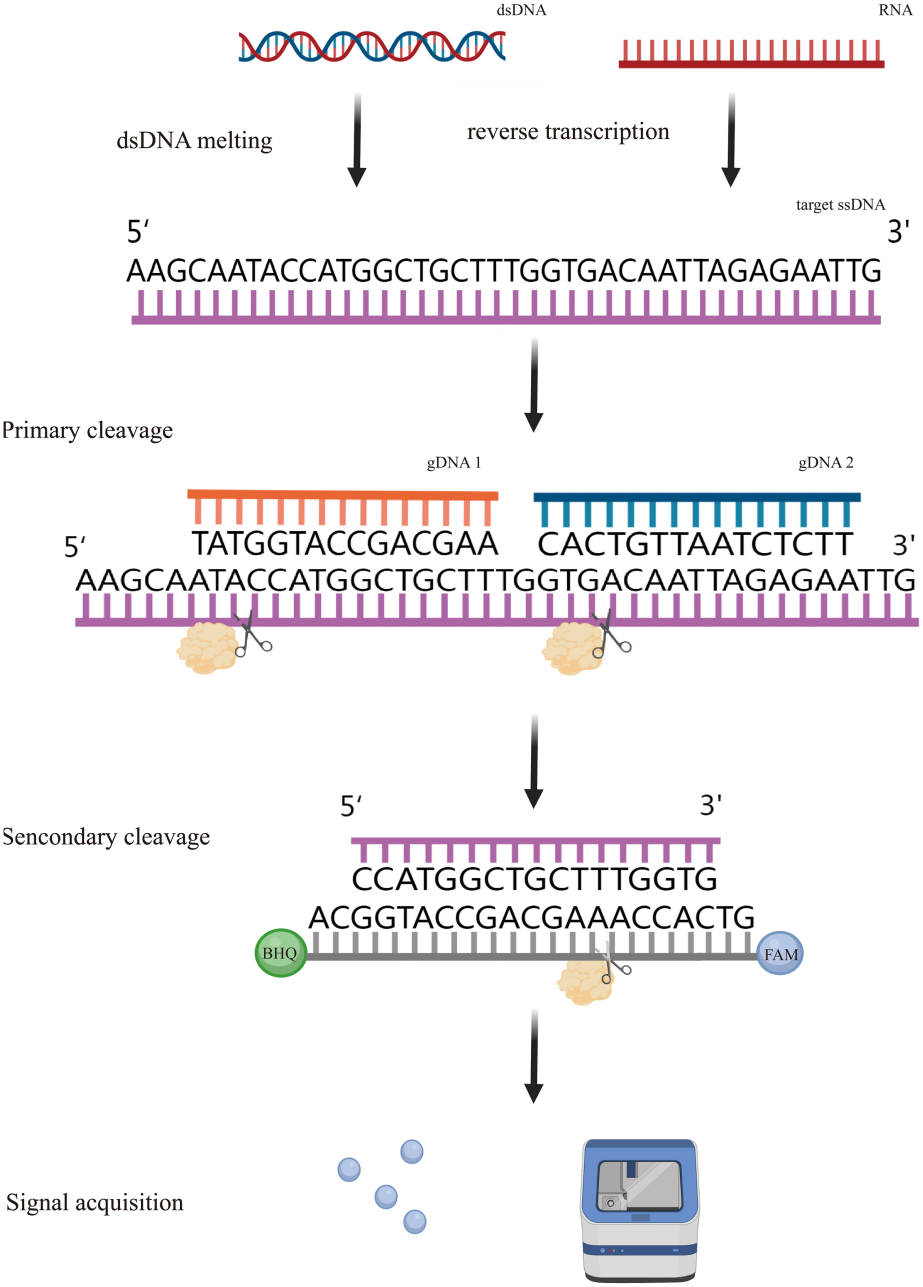
Principle of nucleic acid detection based Argonaute.

Although researchers have invented various nucleic acid detection platforms, the overall principle is similar, as shown in Figure 4. These methods are based on the classical two-step cleavage method of Argonaute. The corresponding primary guide is designed according to the target DNA, and the target is cut by the Argonaute protein to obtain the secondary guide. Then the designed reporter is cut by the Argonaute protein under the guidance of a secondary guide to obtain a fluorescence signal, and in this process, PCR or isothermal amplification can be combined to improve the detection sensitivity. In a study of foodborne pathogens, researchers provided suggestions for developing a one-step cleavage assay called a Novel and One-step cleavage method based on Argonaute by integrating Tag-specific primer extension and Exonuclease I (*Exo* I) (NOTE-Ago) (Li et al., 2023). The amplicons were served as the guide DNA for *Pf*Ago. Consequently, the fluorophore-quencher reporter can be cleaved by *Pf*Ago, leading to alterations in fluorescent intensity. Notably, Argonaute 2 (Ago2) serves as a crucial constituent within the RNA-induced silencing complex (RISC), contributing substantively to a spectrum of physiological phenomena. Conversely, aberrant modulation of Ago2 functionality exhibits a strong association with an array of human maladies, encompassing malignancies. Elucidating the actions of Argonaute 2 has prompted scholars to devise diverse techniques aimed at discerning its operations. These methodologies encompass the utilization of a dual signal amplification-assisted DNAzyme biosensor and a singular-molecule biosensor featuring gold nanoparticles. These innovative approaches have been devised with the intent of diagnosing pathologies linked to Ago2, thereby unveiling promising avenues for extensive clinical applications (Zhang et al., 2018; Wang et al., 2020; Jiang et al., 2022; Zhao et al., 2023). Table 2 provides a comparison of various CRISPR-Cas and Ago-based assays. The SHERLOCK methodology demonstrated proficiency in identifying the Zika virus (ZIKV) and dengue virus (DENV). This accomplishment resulted in a remarkable level of sensitivity at the attomolar scale (10^−18^ M) along with the capacity to discern single-base distinctions. The maturity of this technique is highly evident, as evidenced by its extensive application in the detection of pathogenic bacteria (Gootenberg et al., 2017). It is evident that the nucleic acid detection system utilizing Ago protein is comparable to CRISPR in terms of sensitivity. However, its notable advantage lies in not requiring an additional PAM sequence, making it highly applicable. Furthermore, it is worth noting that the majority of detection platforms rely on thermophilic Ago proteins (*Pf*Ago and *Tt*Ago). While these proteins offer high detection sensitivity, they necessitate the use of PCR or other heating equipment, thereby restricting their portability. Therefore, the CRISPR based detection system is more widely used because it can be detected at room temperature. Consequently, there is significant potential in developing nucleic acid detection technology based on mesophilic Ago proteins, as it would provide a highly sensitive alternative without the limitations of requiring extensive heating equipment.

**Table 2:** Comparison of CRISPR-based and Ago-based detection methods.

Detection methods	Nuclease	Operation	Target motif requirement	Guide oligos	Sensitivity	Portable	References
DETECTR	LbCas12a	62°C for 20–30 min; 37°C for 10 min	PAM	gRNA	aM	Yes	Broughton et al. (2020)
SHERLOCK	LwaCas13a	42°C for 25 min; 37°C for 30 min	PAM	crRNA	aM	Yes	Gootenberg et al. (2017)
PAND	*Pf*Ago	55°C for 10 min; 45 PCR cycles; 95°C for 20–30 min	No	gDNA	aM	No	He et al. (2019) and Wang et al. (2021)
PLCR	*Pf*Ago	30 cycles LCR; 70 min	No	gDNA	aM	No	Wang et al. (2021)
USPCRP	*Pf*Ago	70°Cfor 1 s；40 cycles PCR; 40°C for 1 s	No	gDNA	aM	No	He et al. (2021)
MULAN	*Pf*Ago	65°C for 30–40 min; 95°C for 10–15 min	No	gDNA	5–15 copies/reaction	No	Ye et al. (2022)
NAVIGATER	*Tt*Ago	98°C for 10 s; 63°C for 3 min; 72°C for 30 s	No	gDNA	~1copy	Yes	Song et al. (2020)
MAIDEN	*Pb*Ago/*Km*Ago	42°C for 1 h	No	gDNA	nM	Yes	Li et al. (2022)
SPOT (RT-LAMP)	*Pf*Ago	IVT; 63°C for 30 min; 95°C for 5 min	No	gDNA	7.5 copies/reaction	Yes	Xun et al. (2021)
TtAgoEAR	*Tt*Ago	66°C	No	gDNA	aM	Yes	Yuan et al. (2023)
NOTE-Ago	*Pf*Ago	37°Cfor 5 min; 85°Cfor 5 min; 95°C for 45 min	No	gDNA	1 CFU/mL	Yes	Li et al. (2023)

## Prospects for practical applications of Argonaute proteins

By establishing various Argonaute-based nucleic acid detection platforms, we can (Wu et al., 2007) identify disease-causing pathogens for disease diagnosis and (Shamshirgaran et al., 2022) perform single-base mutation detection for pathogen identification and typing (Zhang et al., 2019). Single base mutation testing in human genomic DNA to predict the probability of disease by predicting the risk of single-base mutations (Mojica et al., 2009). Multiplex nucleic acid testing for use in clinical testing. This is based on the fact that argonaute can be used to cut arbitrary nucleic acid sequences to obtain “sticky” ends of the desired length and nucleotide composition compared to traditional restriction endonucleases. In comparison to CRISPR-Cas9, Ago protein functions as a nucleic acid enzyme capable of cleaving target sites. Unlike CRISPR-Cas9, Ago protein is not restricted by the presence of a protospacer adjacent motif (PAM) sequence. By meticulous design of the nucleic acid guiding strand, precise cleavage of arbitrary target nucleic acids can be achieved. Typically utilizing shorter lengths of genomic DNA (ranging from 15 to 24 nucleotides), the synthesis of short gDNA is more cost-effective than longer crRNA. This may potentially facilitate high-throughput guide RNA production, thereby enabling efficient high-throughput genome editing screening. Moreover, the size of pAgo is approximately 75–85 kDa, approximately half the size of Cas9 and Cas12a, which could enhance the efficient delivery of pAgo to the desired host. This provides excellent conditions for its more comprehensive application (He et al., 2019; Kropocheva et al., 2022). In addition, both *Mp*Ago (*Marinitoga piezophila* Argonaute) and *Km*Ago can be used to probe the high-level structure of RNA. The Ago protein uses RNA as a guide and binds complementary target RNA. The target sequence recognized by nucleotide-specific sites, which connects structured RNA to guides loaded with different sequence sites of argonautes, is incubated. Cleavage sites can be detected either directly or by reverse transcription, and differences in the cleavage efficiency of single and double-stranded RNAs can be used to study the high-level structure of RNA (Schirle et al., 2014; Lapinaite et al., 2018; Kropocheva et al., 2021; Liu et al., 2021).

Recently, DNA-PAINT (DNA-Point Accumulation In Nanoscale Topology) applies Argonaute proteins to super-resolution microscopy, allowing structures to be observed beyond the microscopic fraction (Filius et al., 2020). This super-resolution method relies on the binding and unbinding of the DNA imaging strand, and the key to this technique is the rate of DNA binding. Generally, the binding rate of this technique is 10^6^ M^−1^ s^−1^, and it takes several hours to obtain images with a high spatial resolution (5 nm). In contrast, Argonaute proteins can pre-arrange the target region into a helical conformation (Chandradoss et al., 2015; Yao et al., 2015), allowing a double helix to form between the guide nucleic acid and the target nucleic acid sequence, resulting in a binding rate close to approximately 10^7^ M^−1^ s^−1^. *Cb*Ago (*Clostridium butyricum* Argonaute) is employed in this technique. Its well-targeted properties allow Ago-PAINT to generate super-resolution images of diffraction-limited structures as much as 10 times faster than conventional DNA-PAINT, which provides a more favorable tool for visualizing cellular networks during the study of disease development (Cui et al., 2019; Hegge et al., 2019; Filius et al., 2020). In addition, a research team has applied Ago protein to detect microRNAs (miRNAs) and developed a highly reliable miRNA profiling technique, named Ago-FISH (Argonaute-based Fluorescence *In Situ* Hybridization) (Shin et al., 2020). *Tt*Ago, which is more stable under experimental conditions, is preloaded with DNA probes. This method accelerates the targeted binding of DNA probes, maintaining a high specificity while significantly improving the speed of miRNA detection. Similarly, some researchers have used Ago proteins for dynamic observation of transcriptional processes *in vivo*. By fluorescently labeling the Ago protein NRDE-3, the dsRNA was programmed to observe the transcriptional process in the organism. This method avoids the *in vitro* synthesis of antisense RNA and transfer, which makes the whole observation process faster and easier, and provides a new idea for transcriptome engineering (Toudji-Zouaz et al., 2021).

Argonaute proteins also play an essential role in maintaining homeostatic mechanisms with RNA interference. It has been shown that when viral infection occurs in eukaryotes, siRNA can form RNA-induced silencing complexes (RISC) with Ago proteins and direct the degradation of the associated viral nucleic acids to maintain homeostasis in the organism (Lisitskaya et al., 2018; Zhang et al., 2022). This provides new ideas for studying the progress and treatment of diseases.

## Issues and perspectives

Argonaute proteins still have a long way to go from nucleic acid detection technology to a full-fledged molecular diagnostic method. Although some countries have granted emergency use authorizations and Ago proteins have been rapidly developed for use as a product in molecular diagnostics, their future development will continue to require new explorations (Qin et al., 2022). In addition, the activity and stability of the same Ago protein vary in different experiments and must be further investigated and standardized. Detection limits, intra-experimental reproducibility, clinical sensitivity, and diagnostic specificity in the report for nucleic acid testing platforms involving Ago proteins must be further standardized. Currently, fewer Ago proteins can be used to develop nucleic acid detection platforms, and researchers need to mine more Ago proteins with nucleic acid endonuclease activity that can be used in actual detection systems. In recent years, mesophilic pAgos with DNA cleavage activity have been mined, including *Cb* (*Clostridium butyricum*) Ago (Hegge et al., 2019; Kuzmenko et al., 2019), *Lr* (*Limnothrix rosea*) Ago (Kuzmenko et al., 2019), *Se* (*Syne-chococcuselongatus*) Ago (Olina et al., 2020), *Km* (*Kurthia massiliensis*) Ago (Kropocheva et al., 2021; Liu et al., 2021), and pAgos with RNA shearing activity include *Tt* (*Thermus thermophilus*) Ago, *Mp (Marinitoga piezophile)* Ago, *Cp* (*Clostridium perfringens*) Ago (Cao et al., 2019), and *Km* (*Kurthia massiliensis*) Ago (Wang et al., 2008). It has recently been shown that *Mbp* (*Mucilaginibacter paludism*) Ago exhibits RNA cleavage properties under a wide range of temperature conditions and is active against both 14–21 nt 5’P-gDNA and 15–18 nt 5’OH-gDNA, which is similar to the previously discovered *Km*Ago protein that cleaves not only RNA targets but also DNA targets precisely (Liu et al., 2021). This broadly targeted Ago protein provides a new idea to develop a platform for nucleic acid detection with a broader range of applications (Li et al., 2022).

Hence, it is postulated that an amalgamation of diverse fields encompassing the Argonaute system, engineering, microelectronics, and miniaturization holds the potential to amplify both detection throughput and automation. An illustrative instance of this is the isothermal amplification, which stands as a transformative advancement compared to the conventional polymerase chain reaction (PCR), offering augmented adaptability and an innovative array of methodologies for on-site detection applications. Furthermore, the combination of isothermal amplification with the latest CRISPR Cas13 collateral cleavage technology profoundly enhances the precision and rapidity of detection. However, the cleavage activity of mesophilic Ago is much lower than that of thermophilic Ago. Therefore, in order to improve the sensitivity of mesophilic, protein engineering strategy to generate an evolved Ago with higher activity at moderate temperature is necessary. At the same time, the cleavage efficiency and accuracy of different Ago proteases depend on temperature, divalent ions, gDNA terminal phosphorylation, and length. Therefore, we should pay attention to these influencing factors when developing new reliable nucleic acid detection platforms to achieve higher sensitivity and accuracy of nucleic acid detection. The Argonaute protein has shown great potential as a new programmable nucleic acid binding protein to participate in the next generation of molecular diagnostic technologies. Continued innovative research will bring its function to a greater extent.

## Author contributions

ZW: Conceptualization, Writing – original draft, Writing – review & editing, Funding acquisition. LY: Formal analysis, Writing – original draft. WS: Funding acquisition, Supervision, Writing – review & editing. JM: Supervision, Writing – review & editing.

## Funding

This work was supported by grants from Jiangsu Innovative and Entrepreneurial Talent Programme (JSSCBS20211594) and the Changzhou Science and Technology Project (Applied Based Research, No. CJ20220102).

## Conflict of interest

The authors declare that the research was conducted in the absence of any commercial or financial relationships that could be construed as a potential conflict of interest.

## Publisher’s note

All claims expressed in this article are solely those of the authors and do not necessarily represent those of their affiliated organizations, or those of the publisher, the editors and the reviewers. Any product that may be evaluated in this article, or claim that may be made by its manufacturer, is not guaranteed or endorsed by the publisher.
